# Myoban hot spring bathing improves gut microbiota composition and short-chain fatty acid levels: a pilot study

**DOI:** 10.1265/ehpm.25-00304

**Published:** 2025-10-18

**Authors:** Midori Takeda, Jungmi Choi, Shunsuke Managi

**Affiliations:** Urban Institute, Kyushu University, 744 Motooka, Nishi-ku, Fukuoka-shi, Fukuoka 819-0395, Japan

**Keywords:** Hot springs, Gut microbiota, Short-chain fatty acid, *Bifidobacterium*

## Abstract

**Background:**

Although many studies have reported the therapeutic effects of hot spring bathing on various diseases, its influence on healthy individuals is not well understood. Myoban Onsen, a sulfur-rich hot spring in Beppu City, Japan, is traditionally believed to improve skin conditions, relieve fatigue, and promote relaxation. However, scientific verification of these effects, particularly their impact on gut microbiota and related metabolic outcomes in healthy individuals, remains scarce. This study aimed to evaluate the effects of Myoban hot spring bathing on gut microbiota composition and SCFA concentrations in healthy individuals.

**Methods:**

In this study, 16 healthy adult males (n = 16) participated in Myoban hot spring bathing four times over two weeks. Fecal samples were collected before and after the intervention, and 16S rRNA sequencing and gas chromatography-mass spectrometry (GC-MS) were performed to analyze gut microbiota composition and organic acid concentrations. The effects of hot spring bathing were evaluated using the Wilcoxon matched-pair signed-rank test to compare pre- and post-intervention.

**Results:**

After Myoban hot spring bathing, there was a significant increase in beneficial gut bacteria, *Bifidobacterium*, *Blautia*, and *Anaerostipes*, compared to pre-bathing (p = 0.0012, p = 0.0103, and p = 0.0017, respectively). Conversely, significant decreases were observed in *Parabacteroides*, *Alistipes*, and *Oscillibacter* (p = 0.0125, p = 0.0215, and p = 0.0125, respectively). Significant increases in SCFAs, including acetic acid, propionic acid, and butyric acid, were observed after Myoban hot spring bathing (p = 0.0067, p = 0.0125, and p = 0.0302, respectively). These findings suggest that Myoban hot spring bathing may benefit healthy adult males.

**Conclusions:**

This study suggests that Myoban hot spring bathing may improve gut health in healthy males. The observed increases in beneficial bacteria and SCFAs indicate a potential contribution to improved health status through modulation of the gut environment.

**Trial registration:**

Registration number: UMIN000055229, retrospectively registered.

**Supplementary information:**

The online version contains supplementary material available at https://doi.org/10.1265/ehpm.25-00304.

## Introduction

Hot spring bathing has long been considered beneficial to health worldwide. Various health benefits of hot springs have been reported, including the improvement and prevention of hypertension [1–3], relief from pain caused by rheumatism and ankylosing spondylitis [4, 5], and the treatment of skin diseases [6–8]. Additionally, studies have suggested a positive correlation between regular hot spring bathing and mental health [9], as well as an inverse relationship between daily hot spring bathing and depression [10]. These studies suggest that hot spring bathing has beneficial effects on both physical and mental health. However, its underlying mechanisms and modes of action remain unclear. In particular, the effects of hot spring bathing on healthy individuals still require further investigation.

In a recent study, the authors showed that hot spring bathing in healthy adults induces changes in the gut microbiota, which vary depending on the hot spring water’s spa types (categories defined by the amount and concentration of the contained substances) [11]. Myoban (alum) Onsen in Beppu City, Japan, is a strongly acidic sulfur spring (pH 2.3) defined as containing ≥2 mg of total sulfur per kg. This hot spring is characterized by its milky-blue waters and the traditional production of “yunohana” (hot spring deposits) collected from its steam vents. The primary mineral constituent of yunohana is halotrichite, a hydrated sulfate mineral composed of iron and aluminum sulfates [12]. The detailed mineral composition of the spring water is presented in Table 1. This mineral composition is believed to contribute to the unique physicochemical properties and potential therapeutic effects of the spring waters. Myoban Onsen has traditionally been regarded as one of the most potent hot springs in Japan, and previous studies have also reported notable physiological [13] effects associated with bathing in its waters, further supporting its suitability as a research subject.

**Table 1:** Composition of Myoban hot spring water.

**Component**	**mg/kg**
**Ion Content**	
Hydrogen ion (H^+^)	5.0
Sodium ion (Na^+^)	13.2
Potassium ion (K^+^)	6.9
Chloride ion (Cl^−^)	3.0
Hydrogen sulfate ion (HSO_4_^−^)	78.2
Sulfate ion (SO_4_^2−^)	468.4
Ammonium ion (NH_4_^+^)	0.2
Thiosulfate ion (S_2_O_3_^2−^)	1.6
Magnesium ion (Mg^2+^)	3.7
Dihydrogen phosphate ion (H_2_PO_4_^−^)	0.6
Calcium ion (Ca^2+^)	11.3
Strontium ion (Sr^2+^)	0.1
Manganese ion (Mn^2+^)	0.1
Iron ion (Fe^2+^)	4.0
Aluminum ion (Al^3+^)	14.0

**Non-Dissociated and Gas Components**	
Metasilicic acid (H_2_SiO_3_)	160.8
Hydrogen sulfide (H_2_S)	1.0
Carbon dioxide (CO_2_)	86.2
Free hydrogen sulfide (H_2_S)	5.6

The gut microbiota, a complex community of trillions of microorganisms in the human intestines, is crucial in maintaining overall health. It influences digestion, metabolism, immune function, mental health and diseases [14–17]. The gut microbiota helps breaking down complex carbohydrates, fibers, and other indigestible compounds, producing short-chain fatty acids (SCFAs) such as acetic, propionic, and butyric acids [18, 19]. SCFAs serve as an energy source for intestinal cells, modulate inflammation, and strengthen the gut barrier [15]. Additionally, gut microbiota plays a role in lipid metabolism and affect insulin sensitivity, reducing the risk of metabolic disorders such as obesity and diabetes [20]. It has also been reported that the abundance of SCFA-producing bacteria is reduced in individuals with depression [21]. Overall, the gut microbiota has been reported to influence various diseases.

Given these findings, hot spring bathing may influence gut health not only in patients with specific diseases but also in healthy individuals. However, little is known about how bathing in different types of hot springs, particularly those with distinctive mineral compositions, affects the gut microbiota and SCFA production in healthy populations. Myoban Onsen represents a unique sulfur spring with a powerful mineral composition and high acidity, characteristics that may lead to more pronounced effects compared with other sulfur springs. In this study, we aimed to investigate the effects of bathing in Myoban hot spring on gut microbiota composition and SCFA concentrations in healthy adults, hypothesizing that its distinctive chemical profile could induce measurable and potentially beneficial changes in these gut-related parameters.

## Materials and methods

### Subjects and design

**Fig. 1:**
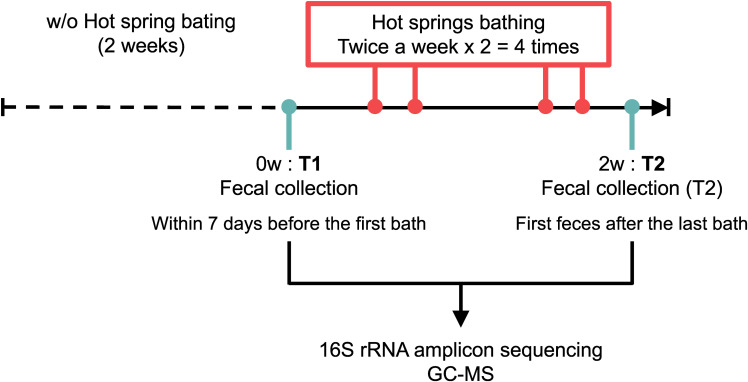
The experimental design. The timeline of the experiment is shown. T1: before trial, T2: after trial.

The study was conducted from January to February 2023. The study population included individuals living in Oita Prefecture in Japan. The inclusion criteria were healthy men between the ages of 18 and 69 without acute and chronic diseases. The exclusion criteria were those on antibiotics. The target number of participants was set at 15. The hot spring bathing was conducted exclusively at “Myoban Yunosato” in Beppu City. Participants were asked not to bathe in any hot springs for two weeks prior to the start of the trial. In the trial, they bathed in the Myoban hot springs four times over two weeks, each for at least 15 minutes (Fig. 1). To prevent unwellness, they were allowed to take breaks during bathing so that the total time spent in the tub exceeded 15 minutes per bathing session.

Participants collected their fecal samples twice, before and after the trial, and sent them to Metagen Inc. (Yamagata, Japan) for measurement. Fecal samples were collected within seven days before the first bathing (Fig. 1, T1 = before trial) and the first feces after the last bathing (Fig. 1, T2 = after trial). The participants were asked to follow their usual diet and lifestyle routines during the trial period.

Fecal samples were analyzed by Metagen Inc. according to the methods described in the subsequent section, and the results were obtained as the relative abundance of gut microbiota and the concentrations of SCFAs. Of the 16 men recruited for the trial, participant ID 15 could not measure due to insufficient sample volume. Therefore, the gut microbiota and short-chain fatty acid results were available for 15 participants.

### Gut microbiome analysis

The gut microbiome profile of fecal samples was analyzed by 16S rRNA amplicon sequencing with the previously described methods [22, 23]. The V1–V2 region of the extracted DNA was amplified by universal primer set 27F-mod (5′-AGRGTTTGATYMTGGCTCAG-3′) and 338R (5′-TGCTGCCTCCCGTAGGAGT-3′). The resulting amplicons were then sequenced in paired-end mode with 600 cycles using the MiSeq platform (Illumina, San Diego, CA, USA). Microbiome analysis was performed using fecal samples collected before and after the study period. 15 subjects were used to identify gut microbiota at the genus level (supplementary Table S1). Because many gut microbial taxa were not consistently detected across individuals, we analyzed the top 20 most frequently occurring genera in all samples (supplementary Table S2).

### Metabolome analysis

Fecal samples were freeze-dried in a VD-800R freeze-dryer (TAITEC) for more than 18 hours. Lyophilized feces were homogenized with 3.0 mm zirconia beads at 1,500 rpm for 10 min using a ShakeMaster^®^ NEO homogenizer (Biomedical Sciences, Tokyo, Japan). Organic acids were measured using a 7890 series gas chromatograph-mass spectrometer (GC-MS, Agilent Technologies, California, USA), as previously described [24].

### Statistical analysis

The Wilcoxon matched pair signed-rank test was used for pre- and post-comparison of relative abundance of gut microbiota and organic acid concentrations. The false-discovery rate (FDR) method was used to correct *p* values for multiple testing, and *q* values were calculated using the Benjamini–Hochberg method. All statistical analyses were performed using R software (version 4.3.1) and Prism 10. Statistical significance was set at *p* < 0.05 and *q* < 0.05.

## Results

The baseline characteristics of the participants in this study are presented in Table 2. Regarding the hot spring bathing schedule, most participants bathed for two days, e.g., on Saturdays and Sundays, and performed two sets. The sample measurements were completed in 15 males; the mean age was 48.9 years, and the mean BMI was 23.2 (Table 2).

**Table 2:** Baseline of the study population.

	**n (%)**	**Mean**	**SD**	**Min.**	**Max.**
**Male**	15 (100)	-	-	-	-

**Age (year)**	-	48.9	7.3	37	65
**BMI (kg/m^2^)**	-	23.2	3.3	17.9	30.6

### Bathing in Myoban hot springs altered specific genera of gut microbiota

**Fig. 2:**
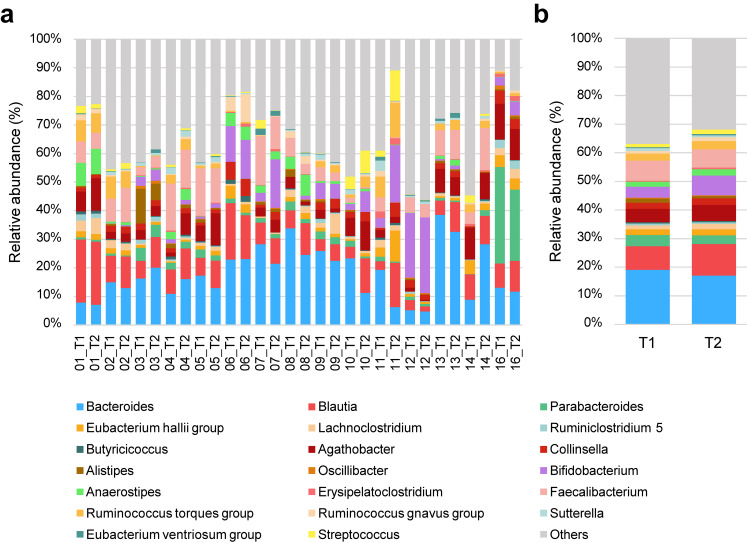
Relative abundance of gut microbiota before and after bathing in Myoban hot springs. (a) One of the stacked bars indicated a fecal sample. The x labels indicate the participant IDs. T1 is the sample before bathing in the hot spring, and T2 is after four baths. (b) The mean values of T1 and T2.

The whole structure of the fecal microbiota of all samples for the 20 identified gut microbiota is shown (Fig. 2a). The mean values of 15 participants before (T1) and after (T2) bathing are also shown in Fig. 2b.

**Fig. 3:**
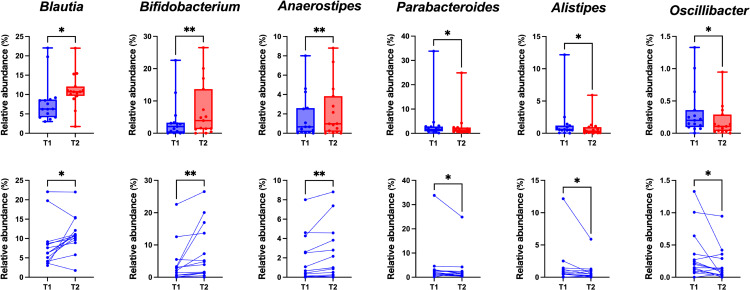
Gut microbiota altered by bathing in the Myoban hot spring. The Wilcoxon matched-pairs signed rank test was used to analyze comparisons before (T1) and after (T2) Myoban hot spring bathing (*p < 0.05, **p < 0.01). (Upper) In the box plots, the box boundary closest to zero indicates the 25th percentile, the line within the box marks the mean, and the box boundary farthest from zero indicates the 75th percentile. Whiskers above and below the box indicate the max and min. Dots indicate the value of each individual. (Lower) Plots corresponding to the same individuals were connected.

The relative abundance of each microbiota was compared before and after the trial. Wilcoxon matched-pairs signed rank tests showed that the relative abundance of *Blautia*, *Bifidobacterium*, and *Anaerostipes* genera was significantly increased after the Myoban hot spring bathing, while *Parabacteroides*, *Alistipes*, and *Oscillibacter* were significantly decreased (Fig. 3, Table 3). Notably, the relative abundance of *Bifidobacterium* was increased to 6.97% compared to 3.85% before bathing (p = 0.0012). *Blautia* and *Anaerostipes* increased from 8.20 to 11.05% and 1.70% to 2.22%, respectively (p = 0.0103 and p = 0.0017) (Fig. 3, Table 3). Meanwhile, *Parabacteroides*, *Alistipes*, and *Oscillibacter* decreased from 4.00% to 3.02%, 1.48% to 0.79%, and 0.33% to 0.18%, respectively (p = 0.0125, p = 0.0215, and p = 0.0125) (Fig. 3, Table 3). Furthermore, q-values were calculated after FDR correction, considering multiple tests. As a result, significant changes were observed in five genera other than *Alistipes*. P-values, q-values, and mean changes are shown in Table 3.

**Table 3:** Pre- and post-comparison of relative abundance of each genus. Genus with significant *p* values are shown in bold. * *p*, *q* < 0.05, ** *p*, *q* < 0.01, Sig. indicates significance.

	***p*-value**	**Sig.**	***q*-value**	**Sig.**	**Mean (%)** **(T1)**	**Mean (%)** **(T2)**	**Mean dif.** **(%)** **(T2–T1)**
*Bacteroides*	0.1205		0.3014		19.0934	17.0358	−2.0577
***Blautia***	**0.0103**	*	**0.0498**	*	**8.2016**	**11.0450**	**2.8434**
***Parabacteroides***	**0.0125**	*	**0.0498**	*	**3.9974**	**3.0155**	**−0.9819**
*Eubacterium hallii group*	0.7615		0.8016		1.9084	2.2097	0.3014
*Lachnoclostridium*	0.3591		0.5986		1.3538	1.8703	0.5165
*Ruminiclostridium 5*	0.6387		0.7997		0.6617	0.5409	−0.1208
*Butyricicoccus*	0.7197		0.7997		0.4493	0.4780	0.0287
*Agathobacter*	0.7148		0.7997		4.7794	5.5326	0.7532
*Collinsella*	0.6698		0.7997		2.0573	2.3987	0.3413
***Alistipes***	**0.0215**	*	**0.0718**		**1.4795**	**0.7861**	**−0.6935**
***Oscillibacter***	**0.0125**	*	**0.0498**	*	**0.3282**	**0.1839**	**−0.1443**
***Bifidobacterium***	**0.0012**	**	**0.0171**	*	**3.8547**	**6.9679**	**3.1132**
***Anaerostipes***	**0.0017**	**	**0.0171**	*	**1.7019**	**2.2229**	**0.5210**
*Erysipelatoclostridium*	0.1189		0.3014		0.4048	0.5342	0.1294
*Faecalibacterium*	0.2439		0.4878		7.0003	6.3332	−0.6671
*Ruminococcus torques group*	0.1909		0.4243		2.4097	2.9418	0.5321
*Ruminococcus gnavus group*	0.2734		0.4972		0.9341	1.3091	0.3750
*Sutterella*	0.5417		0.7739		0.9870	0.6660	−0.3210
*Eubacterium ventriosum group*	0.8926		0.8926		0.4206	0.4337	0.0131
*Streptococcus*	0.4631		0.7125		1.0991	1.6606	0.5615

### Short-chain fatty acids were increased by bathing in Myoban hot springs

**Fig. 4:**
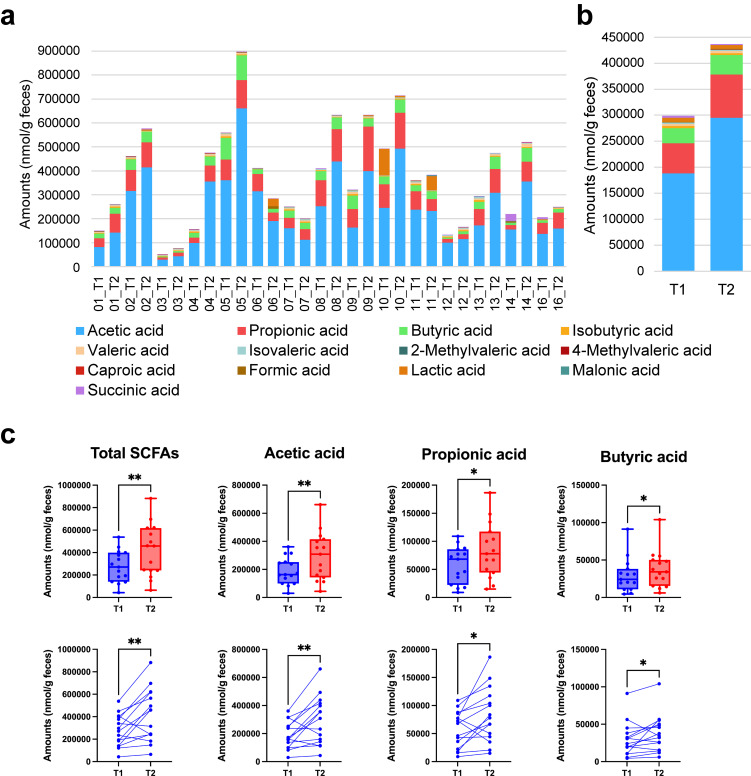
SCFA concentrations are elevated after the Myoban hot spring bathing. (a) One of the stacked bars indicated a fecal sample. The x labels indicate the participant IDs. T1 is the sample before bathing in the hot spring, and T2 is after four baths. (b) The mean values of T1 and T2. (c) The Wilcoxon matched-pairs signed rank test was used to analyze comparisons before (T1) and after (T2) Myoban hot spring bathing (*p < 0.05, **p < 0.01). (c, Upper) In the box plots, the box boundary closest to zero indicates the 25th percentile, the line within the box marks the mean, and the box boundary farthest from zero indicates the 75th percentile. Whiskers above and below the box indicate the max and min. Dots indicate the value of each individual. (c, Lower) Plots corresponding to the same individuals were connected. Total SCFAs includes acetic acid, propionic acid, butyric acid, isobutyric acid, valeric acid, isovaleric acid, 2-methylvaleric acid, 4-methylvaleric acid, and caproic acid.

A total of 14 organic acids were detected in 15 pre- and post-bathing samples. The amount of each organic acid is shown in Fig. 4a and supplementary Table S3. The participants are shown next to each other before (T1) and after four baths (T2). The average values before and after bathing are also shown in Fig. 4b. The results showed that the total amount of SCFAs was significantly increased (p = 0.0084) to 415941.3 nmol/g feces after bathing compared to 275554.9 nmol/g feces before the trial (Fig. 4c, Table 4). Levels of acetic acid, propionic acid, and butyric acid were significantly increased (294963.3, 83439.7, and 37538.3 nmol/g feces) after the trial (T2) compared to before trial (T1) (188473.9, 57791.5, and 29289.4 nmol/g feces) (p = 0.0067, 0.0125, and 0.0302) (Fig. 4c, Table 4). The calculated q-values were only significant for total SCFAs (q = 0.0418) (Table 4).

**Table 4:** Pre- and post-comparison of concentration of detected organic acids. Organic acids with significant *p* values are shown in bold. * *p*, *q* < 0.05, ** *p*, *q* < 0.01, Sig. indicates significance. Total SCFAs includes acetic acid, propionic acid, and butyric acid.

	***p*-value**	**Sig.**	***q*-value**	**Sig.**	**Mean** **(nmol/g feces)** **(T1)**	**Mean** **(nmol/g feces)** **(T2)**	**Mean dif.** **(nmol/g feces)** **(T2–T1)**
**Total SCFAs**	**0.0084**	**	**0.0418**	*	**275554.9**	**415941.3**	**140386.5**
**Acetic acid**	**0.0067**	**	**0.0809**		**188473.9**	**294963.3**	**106489.4**
**Propionic acid**	**0.0125**	*	**0.0809**		**57791.5**	**83439.7**	**25648.2**
**Butyric acid**	**0.0302**	*	**0.1307**		**29289.4**	**37538.3**	**8248.9**
Isobutyric acid	0.3894		0.7232		3734.7	3242.5	−492.1
Valeric acid	0.7197		0.8040		3810.6	4630.6	819.9
Isovaleric acid	0.2769		0.5999		3095.8	2447.3	−648.5
2-Methylvaleric acid	0.6848		0.8040		0.9	0.8	−0.1
4-Methylvaleric acid	0.4973		0.7576		103.1	73.6	−29.5
Caproic acid	0.2769		0.5999		120.5	131.5	11.0
Formic acid	0.8040		0.8040		1117.7	1587.4	469.8
Lactic acid	0.2769		0.5999		7746.4	6960.1	−786.2
Malonic acid	0.7615		0.8040		159.7	314.7	155.0
Succinic acid	0.5245		0.7576		3332.4	1254.7	−2077.7

Based on the above, it is suggested that the increase in *Bifidobacterium*, *Blautia*, and *Anaerostipes* due to Myoban hot spring bathing contributed to the increase in SCFAs. Therefore, Myoban hot spring bathing in healthy adult men may improve the gut environment and overall health.

## Discussions

After bathing in Myoban spring water, *Bifidobacterium*, *Blautia*, and *Anaerostipes* showed a significant increased (Fig. 3 and Table 3). The three bacterial species that increased after bathing are known to have beneficial effects on health. Notably, *Bifidobacterium*, which exhibited the most substantial increase, has been demonstrated to enhance various aspects of human health, including immune defense against infections, when administered as a supplement [25, 26]. Moreover, *Bifidobacterium* is critical in producing bioactive metabolites such as short-chain fatty acids (SCFAs), conjugated linoleic acid, and bacteriocins, contributing to host health [26]. *Blautia* has been reported to exhibit probiotic effects. It improves host health and attenuates metabolic syndrome [27]. Studies have shown a significant inverse correlation between *Blautia* abundance and visceral fat area (VFA) [28] and a reduction in *Blautia* levels in obese children [29]. *Anaerostipes* are also suggested to play a beneficial role in promoting host health [30]. Based on the above, it is suggested that Myoban spring bathing in adult males may induce alterations in the gut microbiota composition, promoting the increase of beneficial bacteria. We confirmed the baseline gut microbiota of the 15 participants with previously reported enterotype distributions in Japanese populations [31]. No participants appeared to show atypical or abnormal microbial patterns, and the overall composition was consistent with that of the general Japanese population.

Mechanistically, warm hydrostatic immersion can enhance parasympathetic (vagal) tone and attenuate Hypothalamic–pituitary–adrenal (HPA) axis stress signaling [32, 33], thereby modulating gastrointestinal motility, secretion, and luminal milieu; these niche shifts may favor saccharolytic, SCFA-producing taxa such as *Bifidobacterium*, *Blautia*, and *Anaerostipes*. In sulfur springs, exposure to dissolved mineral species may also modulate host pathways (e.g., bile-acid and mucosal immune signaling), indirectly shaping microbial communities. Consistent with this, hot-spring therapy with alum, sulfur, and hydrogen sulfide (H_2_S) has been reported to exert diverse physiological effects [13, 34]. We emphasize that these mechanisms are hypothesis-generating and were not directly assessed in this study.

This study showed a significant increase in SCFA concentrations in adult males after Myoban spring bathing compared to before bathing (Fig. 4 and Table 4). Notably, in addition to total SCFAs, the acetic, propionic, and butyric acid levels exhibited a statistically significant increase. These observed changes are likely attributable to the increased abundance of *Bifidobacterium*, *Blautia*, and *Anaerostipes*, as these microorganisms are well-known SCFA producers. Specifically, *Bifidobacterium* ferments carbohydrates via the bifid shunt pathway, generating acetic and lactic acids known to confer health benefits to the host [35]. *Blautia* is recognized for its ability to produce both butyric and acetic acids [28]. Furthermore, *Anaerostipes* has been shown to metabolize inositol stereoisomers into propionic and acetic acids while also functioning as a key butyric acid producer [30, 36, 37]. Given these reports, it is highly plausible that the increase in these three gut microbial genera contributed to the observed rise in SCFA concentrations following Myoban spring bathing. Further research is required to determine whether the increase in SCFA levels induced by hot spring bathing exerts physiological effects, such as alleviating constipation and modulating immune function.

In this study, we observed a significant increase in *Bifidobacterium* after bathing in Myoban (sulfur) hot spring with an increased number of male participants. This result was not observed in our previous study [11]. Compared with our last study [11], the present investigation also differed in several methodological aspects: the number of participants was increased from 10 to 15, only males were included, whereas females had been included previously, and the intervention was conducted at a single facility rather than across two different hot spring sites. These differences may partly explain the discrepancies observed between the two studies. Additionally, baseline variation in gut microbiota among individuals is another potential factor influencing the outcomes. Future investigations should therefore not only consider variables such as sex but also evaluate the effects of hot spring bathing according to individual enterotypes, as this may provide further insight into differential responses.

A limitation of this study is that the findings are based on male participants, thereby constraining the generalizability of the results. Future studies should extend investigations to female participants and individuals with different enterotypes to understand the effects of hot spring bathing on the gut microbiota. Additionally, the study’s generalizability is limited by its relatively small sample size and focus on Japanese adult males. Given that gut microbiota composition is known to vary with age and ethnicity [26, 38], future research should explore these effects in more diverse populations to establish broader applicability. Moreover, the study employed a pre-post comparison design without a control group, which limits the ability to attribute observed changes specifically to Myoban spring bathing. To more rigorously assess the distinct effects of hot spring bathing, future studies should incorporate control conditions, such as bathing in tap water or showering, to distinguish hot spring-specific influences from general bathing effects. Although genus-level changes and alterations in SCFAs were observed, technical limitations of this study prevented the evaluation of alpha- and beta-diversity; therefore, it will be necessary for future research to clarify these potential effects. Despite these limitations, our findings revealed statistically significant and biologically relevant changes. The observed increase in beneficial bacteria and SCFAs following Myoban spring bathing in Japanese adult males constitutes a novel and significant contribution to understanding hot spring-induced alterations in gut microbiota. These findings provide valuable insights into the potential health benefits of hot spring bathing, warranting further investigation through more extensive, more diverse, and controlled studies. Improvements in the gut microbiota composition induced by hot spring bathing may contribute to disease prevention and promote overall health. Future research is expected to understand these effects, highlighting the role of hot spring therapy in enhancing gut health, modulating immune function, and mitigating the risk of lifestyle-related diseases.

In adult males, Myoban hot spring bathing significantly increased beneficial gut microbiota, *Bifidobacterium*, *Blautia*, and *Anaerostipes*, compared to pre-bathing levels. Additionally, a significant elevation in SCFA concentrations was observed, suggesting the potential for positive physiological effects associated with Myoban hot spring bathing. Future research is needed to elucidate how Myoban hot spring bathing influences gut microbiota composition and overall systemic health.

## Supplementary information

**Additional file 1:** **Supplementary Table S1.** List of relative abundance of each gut microbiota before and after hot spring bathing. **Supplementary Table S2.** List of relative abundance of top 20 gut microbiota before and after hot spring bathing. **Supplementary Table S3.** List of organic acids’ concentrations before and after hot spring bathing.

## Declarations

### Availability of data and material

All data generated or analyzed during this study are included in this published article (and its Supplementary Information files).
